# Survey on Ochratoxin A Occurrence in Cured Meat Products in The Netherlands

**DOI:** 10.3390/toxins18060262

**Published:** 2026-06-09

**Authors:** Marta Magdalena Sopel, Hester van den Top, Josipa Grzetic Martens, Monique de Nijs

**Affiliations:** Wageningen Food Safety Research, Wageningen University & Research, Akkermaalsbos 2, 6708 WB Wageningen, The Netherlands

**Keywords:** ochratoxin A, mycotoxin, occurrence, dry-cured meat products, animal-derived food

## Abstract

Ochratoxin A (OTA) is a toxic metabolite produced by fungi, that can be present on various food products, cereals and plant-derived (raw) feed (materials). It was demonstrated that OTA has toxic effects after consumption by both animals and humans. Therefore, the European Food Safety Authority (EFSA) concluded that contribution to human exposure of OTA from (processed) meats should not be ignored. The objective of this study was to assess the occurrence of OTA; thus, data on OTA in cured meats were collected. An in-house validated analytical method using methanol extraction, clean-up with an immunoaffinity columns and LC-MS/MS detection was applied. Quantification was done through the external calibration of standards in solvent using ^13^C_20_ OTA internal standard, with a reporting limit of 0.2 µg/kg and LOQ of 0.04 µg/kg. A total of 50 cured meat products were obtained from Dutch supermarkets. OTA was detected at or above the reporting limit in four samples of cured ham (range 0.30 µg/kg to 79.8 µg/kg) and two samples of sausages (0.2 µg/kg and 0.41 µg/kg). Overall, OTA was detected in twenty samples, and it was concluded that OTA occurred above the LOQ in 40% of cured meats analyzed.

## 1. Introduction

Ochratoxin A (OTA) is secondary metabolite and toxin, primarily produced by fungi of the species *Aspergillus* and *Penicillium*, and may be present in various cereals and cereal products, as well as in food products like coffee, figs, raisins, grapes, liquorice, spices and cocoa. Other forms of ochratoxin may also be encountered, including ochratoxin B (OTB), ochratoxin C (OTC), ochratoxin α (OTα), ochratoxin β (OTβ), (4-R)- and (4-S)-hydroxyochratoxin A (4R-OH OTA and 4S-OH OTA), 4-hydroxy-ochratoxin B (4-OH OTB), and 10-hydroxyochratoxin A (10-OH OTA). However, the occurrence data of these metabolites in food are scarce. Even if there is a concern that these modified forms may contribute to overall exposure when combined, OTA is the most prevalent, studied, and thus relevant form of this group [1,2].

Raw food and feed materials are susceptible to contamination by OTA-producing fungal species during the field and storage period. Additionally, OTA present in feed can be transferred to the bloodstream and tissues of farm animals and eventually reach edible animal tissues. Meat can also be contaminated on the outside with environmental fungi, some of which can produce OTA during curing and storage. Key OTA producers in these environments include *A. alliaceus, A. carbonarius, A. ochraceus, A. steynii, A. westerdijkiae, P. nordicum,* and *P. verrucosum* [3]. However, *P. nordicum* is frequently isolated from specific dry-cured meats [4].

Due to the various contamination pathways, several laboratory sampling approaches can be employed to assess dry-cured meat for OTA contamination. For ham analysis, the fat, skin or rind is removed to sample the inner side, and sampling is typically conducted from the central part of the piece, focusing on the *Semimembranosus* and *Gracilis* muscles [5,6,7,8]. In the case of dry sausages, the non-edible casing is eliminated from the sampling process [9,10,11], and slicing of the product is applied when sausages are intended to be consumed as a whole [12,13,14]. For analysis of the entire product, samples are taken from the middle and both ends [10,15]. Some studies have explored the migration of OTA from the surface into deeper layers of meat products. In these cases, separate analyses were conducted on the casings and the outer and/or inner parts of the product [16,17,18,19]. Additionally, investigations were carried out where samples were taken from the core of each product [20] or from just underneath the casing, which is the edible part where mycotoxins produced on the casings tend to spread the most [21]. To date, there are no standardized methods for sampling meat products within the EU or among member states (MSs) that have established legal limits for OTA in animal-derived products. In this study, we cryogenically milled the entire product for analysis to evaluate consumer exposure.

The European Food Safety Authority (EFSA) has issued several opinions on risks of OTA in both feed and food [22,23,24,25]. In their 2006 opinion, the EFSA evaluated the public health risks associated with OTA in food, describing the accumulation of OTA in kidneys after transfer from food, and identified the kidneys as the organ most susceptible to its toxic effects [23]. Considering the long half-life of OTA in vivo, the EFSA established a tolerable weekly intake (TWI) of 120 nanograms per kilogram of body weight in humans. In 2020, EFSA re-evaluated the toxic effects of OTA based on new evidence [24]. Studies from 2010 onward revealed the mechanism of carcinogenic effects on the kidneys, and thus the need to apply a margin of exposure (MOE) approach [25]. The EFSA’s calculated MOEs for non-neoplastic effects raised health concerns, particularly for infants, toddlers, and other children, especially within the high-consumer groups. Based on exposure assessment data, the EFSA concluded that certain foods, such as preserved meat, ripened cheese, and grain-based products, could significantly contribute to OTA exposure [25].

To minimize human exposure, the European Commission (EC) has established legal limits for OTA in various plant-based food matrices [26]. To address the concern of feed-to-animal tissue transfer, the EC has issued guidance values for OTA in feed (materials) [27]. In accordance with the Regulation (EU) 2017/625 [28], Food Safety Authorities of several MSs implemented monitoring programs for OTA in milk and in tissues from various animal species (typically pig, poultry, bovine, sheep, goat, horse and fish), with a focus on kidneys, muscle and liver. Several MSs also issued national legal limits and guidance values. A guideline value of 1 μg/kg for OTA in pigs’ meat and its derivatives is in place in Italy [29], and Estonia established a legal limit of 10 μg OTA/kg for pig liver [30]. In Denmark, pig kidneys are discarded when the OTA concentration is above 10 μg/kg, the entire carcass is rejected when OTA exceeds 25 μg/kg in the kidneys, and edible offal is thrown away if the OTA concentration in pig kidneys falls between 10 and 25 μg/kg [20,31,32]. Poland established a recommended level of 5 μg/kg for OTA in animal tissues (kidneys, liver and muscles) and 50 and 100 μg/kg in feed for pigs and poultry, respectively [32]. No legal limit exists for OTA in meat (products) in the Netherlands, and no harmonized limits are in place for OTA in animal-derived products in the European Union (EU).

While many methods are described for the analysis of OTA in plant (derived) materials, analytical methods for the quantification of OTA in animal-derived products are scarce. Most analytical methods described in the literature for OTA quantification in meat and sausages use acids or bases to extract OTA from the matrix, with a base–acid liquid–liquid partition to enhance extraction efficiency. Hexane is often used to defat the samples, and extracts are purified using C18 or immunoaffinity columns (IACs) [21,33,34], or by employing QuEChERS-based phase separation [12]. Some confirmatory analyses involve OTA methylation or OTA cleavage (by carboxypeptidase treatment) [6,14,15,35]. For most liquid chromatography coupled with mass spectrometry (LC-MS)-based methods, extensive sample clean-up is not necessary [36,37]. Methods that describe the detection and quantification of OTA in cured meat rely mostly on high-performance liquid chromatography (HPLC) with fluorescence detection (FLD) [7] or LC-MS [9,12,21]. The use of enzyme-linked immunosorbent assays (ELISA) [11,16,38], fluorescence spectroscopy [6] and quadrupole time-of-flight mass spectrometry (QTOF-LC-MS) [6,37] has been described to a lesser extent. The limits of detection (LODs) and limits of quantification (LOQs) depend on the analytical technique; for example, the LOQs for OTA range from 0.025 µg/kg to 50 µg/kg in meat [14,20]. Considering the various limits set by some MSs, the LOQs, as set in Italy, for cured meat should aim at 0.2 µg OTA/kg (0.2 times the legal limit) [10].

In the light of the EFSA opinion of 2020, it is advisable for each MS to conduct screening programs for the presence of OTA in cured meats and cheese. Occurrence data are crucial for proper risk assessment purposes associated with those food categories’ consumption among the whole EU population. The aim of this study was to develop and validate an analytical method at the recommended LOQ (in this study, called LOQ, or reporting limit) and conduct a survey using that method on the occurrence of OTA in cured meat available at supermarkets in the Netherlands.

## 2. Results and Discussion

### 2.1. Fit-for-Purpose Validation

The method was validated for the parameters linearity, specificity, ion ratio, retention time (RT), recovery and repeatability. OTA was detected in the Jamon Iberico sample at 23 µg/kg; therefore, this sample was excluded from calculations (Table 1). OTA was not detected in the other five samples above the target limit of quantification (LOQ) of 0.2 µg/kg. The correlation coefficient of the calibration line was >0.99. The average recovery at the LOQ level was 104.3%, at 1 µg/kg the recovery was 99.2%, and at 5 µg/kg the recovery was 100.5%. The corresponding repeatability was 3.4, 1.6 and 3.1%, respectively. Ion ratio deviations were within ±30% from ion ratios from standards in solvent. No retention time deviations greater than 0.1 min were observed. The LOD was 0.04 µg/kg, based on an S/N ratio of 3. All parameters were within the ranges indicated by the in-house validation plan, as indicated in Section 4.5. Fit-for-Purpose Validation.

#### Additional In-House Validation

**Figure 1:**
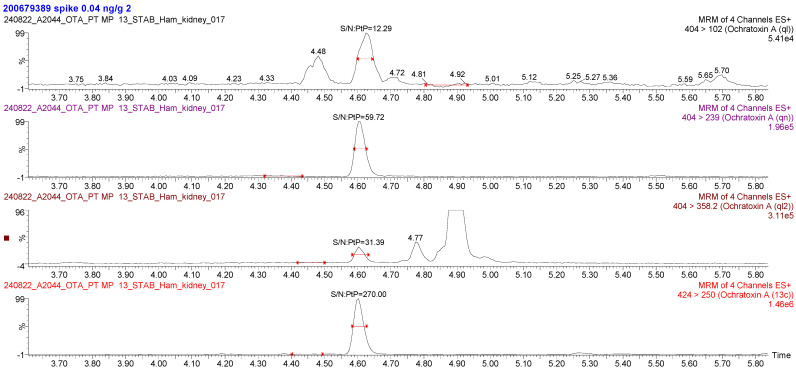
Example chromatogram of Prosciutto di Parma sample spiked at 0.04 µg/kg (ql—qualifying ion, qn—quantifying ion).

Initially, an LOQ of 0.2 µg/kg (the reporting limit) was selected to fulfill the requirement of the LOQ being preferably 0.2 of the ML, based on the Italian legal limit for OTA in meat set at 1 µg/kg, which is the lowest identified among EU member states [29]. The lowest calibration point applied in measurements corresponded to 0.075 µg/kg OTA in the matrix. During the latter real sample analysis, due to the good linearity, it was assumed that the calibration would still be linear below that point. As the LOD of 0.04 µg/kg still fulfilled the requirements of RT and the ion ratio, it was concluded that, after running the spiking experiments, it could become the lower LOQ for research purposes. We then decided to run additional method performance checks at 0.04 µg/kg with extra calibration point at 0.01 µg/kg. The average recovery at 0.04 µg/kg was 94%, with a corresponding repeatability of 3.5%. Thus, in the text, the results are described as above the LOQ when above 0.04 µg/kg and as above the reporting limit when above 0.2 µg/kg. In Figure 1, an example chromatogram of a sample spiked at low LOQ is presented.

### 2.2. Survey Samples

To the best of our knowledge, this is the first study investigating OTA occurrence in cured meat products in the Netherlands. The individual results of the screening of 50 cured meat samples for OTA, presented in Supplementary Table S1, show an incidence of OTA occurrence in 40% of the samples. OTA was detected above the reporting limit in six (12%) of the samples (average 13.6 µg/kg ± 32.4, median 0.41 µg/kg), while traces of OTA (between LOQ and reporting limit) were detected in fourteen samples (28%). The total concentration was 4.14 µg/kg ±17.8 on average, with a median of 0.10 µg/kg (Table 2). Notably, OTA was detected at or above the reporting limit in Jamon de cebo de campo Iberico (0.39 µg/kg), Truffle salami with parmesan cheese (0.41 µg/kg), Chorizo (0.20 µg/kg) and three cured ham samples (0.30, 0.66 and 79.8 µg/kg); thus, four of them originated from whole meat products (dry-cured ham), with two samples from sausages.

In the literature, reports indicate rates of OTA contamination in dry-cured ham of 2% to 100% in samples tested worldwide, with mean concentrations varying from 0.16 (Croatia) to 21.4 µg/kg (Italy), and a maximum detected concentration of 161 µg/kg (Spain) [39]. For dry-cured sausages, the incidence varies from 0 to 100%, with mean concentrations from 0.06 (Italy) to 10 µg/kg (Egypt) and a maximum concentration of 691 µg/kg (Italy). Other dry-cured meat products had an incidence rate of 2% to 33, in the range of 0.07 (Croatia) to 109 µg/kg (Italy, maximum detected concentration). The incidence of positive dry-cured meat samples varied not only among different countries, but also within one country. For example, the incidence varied from 2 to 64% in Croatia and 4.5 to 100% in Italy [39]. Comparing our findings to other results, OTA occurrence in similar dry-cured meat products in the Netherlands fell within the low range of concentrations in previously described reports, with the occasional elevated contamination of one cured ham sample. Similar variability was reported in other countries where market surveys were conducted [40].

OTA in cured meats may originate from contaminated animal feed or be produced by surface fungi during curing and then migrate into the product [16,41]. In the case of processed meat products such as sausages, OTA can be added when using contaminated ingredients such as spices for meat seasoning [4,17,42]. OTA concentration can further increase during curing due to water loss. The varying OTA concentrations in meats across different countries were linked by the authors to factors such as production technology, the climate of the producing region, or the detection method used. In the current study, only one sample of dry-cured ham was found to be contaminated with a significant concentration of 79.8 µg/kg, which could not be neglected. During the sample preparation, the outer fat layer was removed before homogenization, and an entire portion of approximately 1 kg was cryogenically milled. To determine whether the contamination originated from external environmental factors, spices, or the meat itself, further research on the OTA concentration at different depths of the product would be necessary. However, this was beyond the scope of the study. The only information we may provide is that all four cured ham samples, each weighing about 1 kg, came from the same batch. Unfortunately, we do not know from which specific area of storage room they were taken, making it impossible to conclude whether the contamination resulted from the storage or production environment or from the meat itself. Therefore, it is essential for competent authorities to establish sampling schemes and control measures to reduce the risk of OTA exposure in the general public when consuming these products.

## 3. Conclusions

A straightforward LC-MS/MS-based method for the quantification of OTA in cured meat was developed and successfully validated in-house at a research LOQ and reporting limit of 0.04 and 0.2 µg/kg, respectively, for cured meat and kidneys. The method was found to be fit for purpose. The results of the survey of 50 cured meat samples obtained in the Netherlands revealed that 40% of the cured meat products were contaminated with OTA. In general, the levels were low, but a relatively high level of incidental contamination cannot be excluded. Cured meat products may be considered a potential low-level but frequent dietary source of OTA.

## 4. Materials and Methods

### 4.1. Chemicals and Reagents

The analytical standards of OTA and ^13^C_20_-OTA both at 10 µg/mL in acetonitrile were purchased from Romer Labs GmbH (Tulln, Austria). Acetonitrile and methanol were obtained from Biosolve BV (Valkenswaard, The Netherlands), acetic acid and phosphate-buffered saline (PBS) from Merck Life Science NV (Amsterdam, The Netherlands), sodium bicarbonate from Merck, Ultrapure water for LC-MS and n-hexane from ActuAll (Oss, The Netherlands). Water was purified using a Milli-Q IQ 7000 purification system with a minimal resistance of 18.2 MΩ/cm (Etten-Leur, The Netherlands). Ochraprep^®^ immuno affinity columns were purchased from R-Biopharm AG (Arnhem, The Netherlands).

### 4.2. Samples

A total of 50 samples of cured pork meat were purchased from local supermarkets in the Netherlands in the period of February until June 2023 (see Table 1). The samples were stored in a freezer at −18 °C until analysis. Thirty-five samples were packages of sliced product of about 100 g each, 11 were sausages, and four were cured ham legs of 1 kg each.

For 46 samples, the whole portion of each sample was cryogenically milled before the analysis to ensure sample homogeneity. For the four ca. 1 kg ham samples, the skin and fat were removed before cryogenic milling with liquid nitrogen (samples 47–50, Table 1).

### 4.3. Extraction and Clean-Up

The validated method was used for the analysis of the samples (EURLMP-method_016 v1, 2023 [43]). A 2 g aliquot of sample was weighed in a Greiner tube and spiked with 20 µL of 0.1 µg/mL ^13^C_20_-OTA internal standard (IS). Water was added (3 mL), and the sample was left to rest for 15 min. A volume of 7 mL of methanol and 30 mg of sodium bicarbonate were added, and the samples were mixed by ultra-turrax (IKA-Werke GmbH & Co. KG, Staufen, Germany) for 30 s. The extracts were defatted by adding 3 mL of *n*-hexane and placed in the mechanical shaker head-over-head (Heidolph, Schwabach, Germany) for 30 min. Samples were centrifuged (DJB Labcare Ltd., Newport Pagnell, UK) at 3600 rpm for 10 min, and the upper hexane layer was discarded. The remaining extract was filtered over a fluted filter (Schleicher & Schuell BioScience, Keene, NH, USA). An aliquot of 2 mL of the extract was transferred to a 50 mL polypropylene centrifuge tube, diluted to a total of 25 mL with PBS buffer, and mixed for 30 s on a vortex mixer (IKA-Werke GmbH & Co. KG, Staufen, Germany). The total sample was loaded onto the IAC column and passed by gravity, and the columns were rinsed with 3 mL of water. After applying 3 mL of methanol, a few drops were slowly passed through the column, and the column was rested for 15 min. OTA was eluted from the column by letting the methanol pass, after which the extracts were evaporated to dryness (Biotage, Uppsala, Sweden) under a stream of nitrogen at 55 °C. The dry residue was reconstituted in 200 µL of 50% methanol/water (*v*/*v*) with 0.1% acetic acid and vortex mixed for 1 min. The final extract was transferred to a total recovery vial (Thermo Scientific™, Waltham, MA USA) and stored (if needed) at −18 °C until LC-MS/MS analysis.

### 4.4. LC-MS/MS

All LC-MS/MS measurements were performed using an Acquity UPLC grade system Waters, Milford, MA, USA) equipped with an autosampler, two gradient pumps and a temperature control oven. The system was coupled to a Xevo TQ-XS mass spectrometer (Waters, Milford, MA, USA) equipped with an electrospray ionization (ESI) source, operated in multiple reaction monitoring with positive ionization mode. Cone voltage was set at 20 V, desolvation gas temperature at 500 °C, source block temperature at 150 °C, and the argon collision gas flow at 14 mL/min. OTA was retained on a Waters Acquity UPLC BEH C18 (1.8 µm, 2.1 × 100 mm) analytical column (Waters, Milford, MA, USA), controlled at 40 °C, with a flow rate of 0.4 mL/min. The mobile phase consisted of 0.1% acetic acid in water (A) and 0.1% acetic acid in 95% acetonitrile in water (B). The gradient started at 90% A/10% B, held for 1 min, and changed linearly to 0% A/100% B in 6 min, which was held until 7.7 min. The gradient changed back to the starting conditions of 90% A/10% B within 0.3 min and was held from 8 till 8.5 min. The total run time was 8.5 min. The *m*/*z* ions monitored for OTA were 404.0/239.0 (eV 25), 404.0/102.0 (eV 45), 404.0/358.2 (eV 16) and for ^13^C_20_-OTA 424.0/250.0 (eV 25).

### 4.5. Fit-for-Purpose Validation

The method was validated for linearity (deviation of back-calculated concentration from true concentration ≤ ±20%), specificity (≤30% of reporting limit of 0.2 µg/kg), ion ratio (deviation ≤ 30%), retention time (deviation not more than ±0.1 min), recovery (70–120%), and repeatability (RSDr ≤ 20%) as described by the criteria from SANTE/11312/2021 [44]. Each validation sample was analyzed before and after spiking. Matrix effects (MEs) were compensated by normalization using an IS of ^13^C_20_ OTA. Linearity was checked within the whole calibration curve range of 0.15–22.5 ng/mL (corresponding to 0.075–11.25 µg/kg in matrix) for fit-for-purpose validation and of 0.02–0.75 ng/mL (corresponding to 0.01–0.375 µg/kg) for additional performance checks of the LOQ. The IS, ^13^C_20_-OTA, was added to all samples and to calibration standards at concentrations of 1.5 µg/kg and 3 ng/mL, respectively. To verify the method’s specificity, the average response of blank matrix samples for each group of food commodities in the expected RT for OTA were compared with the average response from the spiked samples. Recovery samples were prepared by spiking different blank cured meat samples (Serrano ham Consorcio 12+, Coppa di Parma, Fuet, Prosciutto di Parma, Jamon Iberico, Salami classic—mini sticks) at three levels, 0.2, 1 and 5 µg OTA/kg, at six-fold with concurrent addition of IS before the extraction. For the additional performance checks of the LOQ, the recovery samples were used to calculate the method repeatability. The LOD is a concentration of sample still conforming to the criteria for ion ratio and retention time.

### 4.6. Data Evaluation

Mass spectrometric data were processed using Masslynx 4.2 software (Waters, Milford, MA, USA). Peak areas normalized to the IS were used for quantification. Concentrations of OTA in meat were determined through multi-level calibration using the solvent standards. Matrix effects were compensated through normalization of analytes’ response to the isotope-labeled IS response. The results were expressed in µg/kg.

### 4.7. Quality Control

For quality control, for each batch of samples analyzed, recovery samples spiked at 0.3 µg OTA/kg were added. Ion ratio and retention time deviations from the calibration line were calculated. QC samples showed acceptable performance, with average recoveries of 118%, thus being within the 70–120% criteria, with precision at 12.8% (criteria below 20%), and ion ratio deviations were within ±30% from the ion ratios from standards in solvent. No retention time deviations greater than 0.1 min were observed relative to solvent standards. Back-calculated concentrations from calibration lines did not deviate more than ±20%.

## Figures and Tables

**Table 1:** Individual results for spiking experiments.

Sample No	Concentration in Unspiked Sample, µg/kg	Results Corrected for Natural Contamination	Recovered Concentrations (µg/kg), Recoveries (%), and Corresponding Repeatability (RSDr, %) at Fit-for-Purpose Validation		
			At 0.2 µg/kg	At 1 µg/kg	At 5 µg/kg
1	0.14	YES	0.211	0.9733	5.002
2	0.07	YES	0.206	0.996	5.008
3	0.06	YES	0.207	1.016	4.971
4	<0.04	NO	0.219	0.992	4.859
5	23.04	No result			
6	0.04	YES	0.200	0.984	5.277
		Recovery average	104.3	99.2	100.5
		RSDr	3.4	1.6	3.1

**Table 2:** Summary results of the survey on OTA in cured pig meat products.

Product Category	Number of Samples	Samples ≤ LOQ (0.04 µg/kg)	Samples Reporting Limit ≥ LOQ (0.04 µg/kg)	Samples ≥ Reporting Limit (0.2 µg/kg)	Mean Concentration ± SD (µg/kg)
Dry-cured ham	*n* = 17	*n* = 10, 59%	*n* = 3, 18%	*n* = 4, 24%	11.6 ± 30.1
Salami and fermented sausages	*n* = 14	*n* = 9, 64%	*n* = 4, 29%	*n* = 1, 7%	0.14 ± 0.15
Other fermented sausages	*n* = 10	*n* = 3, 30%	*n* = 6, 60%	*n* = 1, 10%	0.08 ± 0.07
Whole muscle cured meats	*n* = 4	*n* = 0, 0%	*n* = 4, 100%	*n* = 0, 0%	<0.04
Other products	*n* = 5	*n* = 4, 80%	*n* = 1, 20%	*n* = 0, 0%	0.18 ± 0.00
Total	*n* = 50	*n* = 30, 60%	*n* = 14, 28%	*n* = 6, 12%	4.1 ± 17.8

## Data Availability

The original contributions presented in the study are included in the article; further inquiries can be directed to the corresponding author.

## Supplementary Materials

The following supporting information can be downloaded at: https://www.mdpi.com/article/10.3390/toxins18060262/s1, Table S1: Individual results of the survey on OTA in cured pig meat products.
