# Supplementary material for: Survey on Ochratoxin A Occurrence in Cured Meat Products in The Netherlands
Source: Toxins (Basel). 2026 Jun 9;18(6):262. doi: 10.3390/toxins18060262 (PMC13307899; doi:10.3390/toxins18060262)
Supplement: Supplementary file 1 [file toxins-18-00262-s001.zip › toxins-4281633-supplementary.pdf]

Table S1. Individual results of the survey on OTA in cured pig meat products.

Sample ID	Product	Sample weight (g)	Description on the package material, if available	OTA (µg/kg)
1	Serrano ham Reserva	103	Raw, dried, 14 months ripened ham	<0.04
2	Italian Ham	107	Raw, dried, ripened ham	<0.04
3	Coburger Ham	118	Raw, dried, ripened ham	<0.04
4	Plato de tapas	100	Serrano ham, choriso, lomo	0.18
5	Salame al finocchio	100	Salami with fennel	<0.04
6	Jamon Serrano Gran Reserva	86	Minimum 16 months ripened	<0.04
7	Serrano Gran Reserva	100		<0.04
8	Salame Felino IGP-BGA	100		0.04*
9	Prosciutto di Parma	100		0.07*
10	Spinata Romana con pepe	100	Salami with black pepper	<0.04
11	Spinata Romana	107	Minimum 2 months ripened	0.11
12	Wine cervelat	150		<0.04
13	Capocollo Stagionato	100		<0.04
14	Lonzino Stagionato	100		<0.04
15	Jamon Serrano GTS	100		<0.04
16	Prosciutto Crudo	70		<0.04
17	Pancetta	80		<0.04
18	Prosciutto di Parma	90		<0.04
19	Spanish salami	100		<0.04
20	Salami Milano	100		0.08
21	Schwarzwalder schinken	100	Raw, smoked ham	<0.04
22	Coppa Tradition tapa	120	Raw, dried	<0.04
23	Spinata Romana	120	Fermented, dried pork meat	<0.04
24	Salami Milano	120	Fermented, dried pork meat	<0.04
25	Prosciutto Crudo	90	Dried ham, minimum 10 months ripened	<0.04
26	Salame Milano	80	Minimum 2 months ripened	<0.04
27	Prosciutto di Parma	80	Minimum 16 months ripened	0.12
28	Hungarian salami	79	100 days ripened	<0.04
29	Jamon de cebo de camptio iberico	70	24 months ripened, 50% Iberian breed	0.39
30	Salami Italiani alla Cacciatora	100		0.05*
31	Fuet Catalan	120	Minimum 2 months ripened	<0.04
32	Spinata Romana	80		<0.04
33	Spanish Lomo	70	Dried pork loin	<0.04
34	Longaniza Iberica	120	Dried sausage	<0.04
35	Fuert sausages	140	Pork and beef product	0.05*
36	Spanish besos de Fuet	172	Dries sausages	0.04*
37	Truffles salami	120	With parmesan cheese and black truffles	0.41
38	Saucisson aux Noisettes	200	Dried sausage	0.05*
39	Choriso	200	Dried sausage from Spain	0.20
40	Baton pur porc	225	Dried sausage from France	<0.04
41	Spanish salami	250	Dried sausage from Spain	<0.04

42	Saucisson noisettes	200	Dried sausage from France	<0.04
43	Longaniza de pages	200	Dried sausage from Spain	0.04*
44	Picollini	170	Dried sausage from Belgium	0.04*
45	Baton du porc	250	Dried sausage	<0.04
46	Biologic fuet	150	Dried sausage	0.17
47	Dried ham	1180	Dried ham, 9 months ripened	79.8
48	Dried ham	1216	Dried ham, 9 months ripened	0.30
49	Dried ham	1222	Dried ham, 9 months ripened	0.66
50	Dried ham	1290	Dried ham, 9 months ripened	0.04*

At the time of the measurements: Limit Of Detection (LOD) = 0.04 μg/kg, verified later in additional methods performance checks as low LOQ; Reporting Limit = target LOQ = 0.2 μg/kg; Lowest calibration level = 0.075 μg/kg for sample measurements, and 0.01 μg/kg;

* extrapolated indicative concentrations (based on the assumption that the relationship between concentration and signal remains linear beyond the lowest calibrator)
